# Sexual Health Assessment Is Vital to Whole Health Models of Care

**DOI:** 10.2196/36266

**Published:** 2022-07-28

**Authors:** Alex Uzdavines, Drew A Helmer, Juliette F Spelman, Kristin M Mattocks, Amanda M Johnson, John F Chardos, Kristine E Lynch, Michael R Kauth

**Affiliations:** 1 South Central Mental Illness Research, Education, and Clinical Center Michael E. DeBakey VA Medical Center Houston, TX United States; 2 Center for Innovations in Quality, Effectiveness, and Safety Michael E. DeBakey VA Medical Center Houston, TX United States; 3 Department of Medicine Baylor College of Medicine Houston, TX United States; 4 VA Connecticut Healthcare System West Haven, CT United States; 5 Yale School of Medicine New Haven, CT United States; 6 VA Central Western Massachusetts Healthcare System Leeds, MA United States; 7 University of Massachusetts Medical School Worcester, MA United States; 8 VA Cheyenne Medical Center Cheyenne, WY United States; 9 VA Palo Alto Health Care System Palo Alto, CA United States; 10 Stanford University Palo Alto, CA United States; 11 VA Informatics and Computing Infrastructure VA Salt Lake City Health Care System Salt Lake City, UT United States; 12 Department of Internal Medicine University of Utah School of Medicine Salt Lake City, UT United States; 13 LGBTQ+ Health Program Patient Care Services Veterans Health Administration Washington, DC United States

**Keywords:** sexual health, sexual health assessment, veteran, health equity, health assessment, whole health model, communication, communication barrier, technological barrier, health care, sexuality, sexual orientation, gender identity, sex, gender, model, care, barrier, well-being, comfort, assessment, EHR, electronic health record, quality, equity

## Abstract

Sexual health is the state of well-being regarding sexuality. Sexual health is highly valued and associated with overall health. Overall health and well-being are more than the absence of disease or dysfunction. Health care systems adopting whole health models of care need to incorporate a holistic assessment of sexual health. This includes assessing patients’ sexual orientation and gender identity (SOGI). If health systems, including but not limited to the Veterans Health Administration (VHA), incorporate sexual health into whole health they could enhance preventive care, promote healthy sexual functioning, and optimize overall health and well-being. Assessing sexual health can give providers important information about a patient’s health, well-being, and health goals. Sexual concerns or dysfunction may also signal undiagnosed health conditions. Additionally, collecting SOGI information as part of a sexual health assessment would allow providers to address problems that drive disparities for lesbian, gay, bisexual, transgender, queer, and similar minority (LGBTQ+) populations. Health care providers do not routinely assess sexual health in clinical practice. One barrier is a gap in communication between patients and providers. Providers cite beliefs that patients will bring up sexual concerns themselves or might be offended by discussing sexual health. Patients often report an expectation that providers will bring up sexual health and being comfortable discussing sexual health with their providers. Within the VHA, the lack of a sexual health template within the electronic health record (EHR) adds an additional barrier. The VHA’s transition toward whole health and updates to its EHR provide unique opportunities to integrate sexual health assessment into routine care. We highlight system modifications to address this within the VHA. These examples may be helpful for other health care systems interested in moving toward whole health. It will be vital for health care systems integrating a whole health approach to develop both practical and educational interventions to address the communication gap. These interventions will need to target both providers and patients in health care systems that transition to a whole health model of care, not just the VHA. Both the communication gap between providers and patients, and the lack of support within some EHR systems for sexual health assessment are barriers to assessing sexual health in primary care clinics. Routine sexual health assessment would benefit patient well-being and present an opportunity to address health disparities for LGBTQ+ populations. Health care systems (ie, both the VHA and other systems) can overcome these barriers by implementing educational interventions and updating their EHRs and back-end data structures. VHA’s expertise in developing and implementing health education interventions and EHR-based quality improvements may help inform interventions beyond VHA.

## Introduction

Most people, including veterans (individuals who have served in the Armed Forces, regardless of combat exposure, and who are no longer on active duty after receiving an honorable discharge from military service), are sexually active and value their sexuality [1]. In a US study, 50% of sexually active men and 40% of women rated sexual health as highly important; and self-reported health was closely correlated with perceived importance of sexual health [2]. The World Health Organization defines *sexual health* as:

...a state of physical, emotional, mental and social well-being in relation to sexuality; (...) not merely the absence of disease, dysfunction or infirmity. Sexual health requires a positive and respectful approach to sexuality and sexual relationships, as well as the possibility of having pleasurable and safe sexual experiences. [3].

*Sexuality* is defined as:

...a central aspect of being human throughout life and encompasses sex, gender identities and roles, sexual orientation, eroticism, pleasure, intimacy and reproduction. [3].

### Benefits of Sexual Health and Problems Associated With Disruptions in Sexual Health

Sexual intimacy is meaningful to people and may function as a buffer against chronic stress, depression, and suicidal ideation [3-6]. Conversely, disruptions in sexual intimacy may be a further source of stress that exacerbate existing difficulties. Veterans, especially women and sexual and gender minority veterans, experience a high rate of disruptions in healthy sexual intimacy. This is due to premilitary trauma, high rates of military-related injuries, multiple and often comorbid chronic illnesses (eg, vascular disease, obesity, depression, posttraumatic stress symptoms, substance use disorders, and tobacco use), and medication side effects that interfere with sexual desire and functioning [5,7-11]. While Veterans are often exposed to risk factors that disrupt sexual health during military service, many of these risk factors contribute to sexual dysfunction in the general population as well. Despite this, sexual health is often overlooked in clinical practice, both in the Veterans Health Administration (VHA) and other health care systems, unless the patient voices concerns [2,12].

### Sexual Health Is Important to Whole Health

Sexual health has recently been acknowledged as an integral part of holistic conceptions of human health. An extensive report from the National Academies of Science, Engineering, and Medicine framed the importance of paradigmatic change in terms of sexually transmitted infection (STI) prevention [13]. They argued that the abstinence and disease-based models of STI prevention have largely failed to control STIs in the United States, and there is evidence that a sexual *health* framework is more likely to succeed. We extend their argument; shifting toward a sexual health model is important for more than STI prevention. As medical systems embrace whole health (defined as a focus on the multiple components of well-being based on their values, needs, and goals, and not simply the treatment of illnesses, injuries, and disabilities [14,15]) models of care, they will need to include sexual health or risk excluding an important aspect of their patients’ overall health.

## Benefits of Assessing Sexual Health

As health care systems transition toward whole health approaches to care, it will be essential that providers within these systems know what matters to their patients. This includes a basic understanding of a patient’s values, goals, and overall identity, including their sexual orientation and gender identity (SOGI). This may be particularly important for people with lesbian, gay, bisexual, transgender, queer, and similar minority (LGBTQ+) identities whose values, goals, and sexuality may be overlooked. In addition to helping providers better understand their patients’ values, sexual health assessment provides information that may not come out during a standard clinical assessment.

### Correlation With Other Health Outcomes

Assessing sexual health can give providers important information about a patient’s overall health and well-being; sexual concerns or dysfunction may signal unknown or undiagnosed health conditions. For example, a large meta-analysis demonstrated a correlation between erectile dysfunction in men and increased risk of negative cardiovascular outcomes and all-cause mortality [16]. In addition, sexual health assessment provides an opportunity to discuss treatment side effects such as drug-induced sexual dysfunction, which is common with the use of antihypertensives and antidepressants [17].

Conversely, providers can anticipate sexual health dysfunction when their patients have diagnoses known to impact sexual health or function. For example, sexual health concerns are associated with obesity, diabetes, obstructive sleep apnea in men, and metabolic syndrome in women [18-20]. Discussing this association with patients is one avenue providers can use to introduce the concept of sexual health and potentially motivate patients for behavioral change. In the absence of discussing sexual health, providers may miss the larger patient-centered picture and ignore the interrelationship between sexual health and overall health. For LGBTQ+ patients, this relationship between sexual health and overall health may be especially important.

### Decreasing Disparities for LGBTQ+ Patients

There is substantial evidence that LGBTQ+ people experience worse physical and mental health outcomes relative to non-LGBTQ+ people [21-26], likely related to sexual and gender minority stress. Minority stress theory predicts that LGBTQ+ people will experience worse mental and physical health outcomes due to chronic stress caused by anti-LGBTQ+ victimization and stigma in society as well as internalization of these biases [25,26]. For LGBTQ+ veterans, this pattern holds [27], especially in relation to sexual health [28], and may be exacerbated by having served in the military under “Don’t Ask, Don’t Tell” [29] and increased exposure to sexual assault and harassment while in service [30-32]. Health care systems implementing policies to normalize the collection of SOGI data in routine clinical practice would help clinicians identify patients at increased risk for poor general and mental health outcomes due to chronic exposure to sexual and gender minority stress.

### Enhancing Whole Health and Patient-Centered Approaches

The VHA has committed to a whole health approach to care where providers partner with veterans to help them achieve their health goals [15]. Although the VHA’s whole health approach covers most aspects of well-being, it stops short of explicitly discussing sexual health. This approach underemphasizes the importance of good sexual functioning and healthy sexual intimacy on patient values or goals. Incorporating sexual health in whole health materials designed to prompt patient-provider communication would signal to providers and patients that these topics can be openly discussed.

## Addressing Barriers to Sexual Health Assessment

### The Gap Between Providers’ and Patients’ Beliefs About Sexual Health Assessment

#### Sexual Orientation and Gender Identity

Understanding core features of patients’ identities is essential in building trust between patient and provider, and identifying their values. Yet assessment of SOGI—critical first steps for providers conducting sexual health assessments and developing a holistic understanding of their patients’ identities—is not routine despite recommendations from the National Academy of Medicine and Joint Commission [33,34].

Providers often cite lack of time and training, uncertainty around how SOGI affect health, and a belief that patients will bring up their SOGI as reasons for not proactively asking for this information [35-38]. In one qualitative study of primary care providers (n=25), participants stated that, given the limited time allotted for appointments, asking about SOGI would take time away from asking, or bias their assumptions, about their patients’ sexual behaviors and current primary and secondary reproductive anatomy (ie, anatomical inventory; for more information see Grasso et al [39]) [40]. Providers also believed that asking about SOGI might lead to patient discomfort.

In contrast, studies find that most patients are comfortable discussing their SOGI with their health provider. In the recent EQUALITY study that included a sample of 429 emergency department providers, roughly 80% of providers worried that asking patients about SOGI would offend them; however, only 9% to 11% of patients (n=1516) thought they might be offended. By contrast, about half of patients and 74% to 80% of providers believed that knowing a patient’s SOGI would give them a better understanding of the whole person [41,42]. In a study of 304 primary care patients drawn from 4 community health settings, investigators found that about 90% of patients think their SOGI information is important for the provider to know and only around 10% think they would refuse to answer SOGI questions on hospital registration forms [43].

There is also evidence that people overwhelmingly answer SOGI questions. An analysis of the 2014 Behavioral Risk Factor Surveillance System data for the 20 states that included a SOGI module found that both veterans (n=22,587) and nonveterans (n=146,475) had very low rates of refusal in answering SOGI questions (1.5% and 1.9%, respectively). These data also indicated that veterans were more likely to respond to SOGI questions than nonveterans [44]. These behavioral data reinforce that routine SOGI assessments are an important aspect of patient-centered care.

An important nuance to this discussion is that due to discrimination in society and the military, some LGBTQ+ veteran patients worry about disclosing their sexual orientation or gender identity to VHA health care providers [45]. Many lesbian and bisexual women veterans have experienced discrimination, rejection, or poor care after disclosing their sexuality to providers and may avoid conversations regarding sexual identity [46]. These experiences may reinforce discrimination and traumas encountered during military deployments due to their perceived sexual orientation [31]. Worries stemming from discrimination may lead to delays in seeking health care, compounding poor health outcomes for lesbian and bisexual women veterans. The VHA and providers must demonstrate that they are welcoming and affirming of LGBTQ+ veterans [47], and they can do this, in part, by normalizing SOGI and sexual health assessment for all patients.

#### Taking a Sexual Health History

The same patterns hold for assessing sexual health more broadly. Namely, providers feel uncomfortable about assessing sexual health without the patient bringing up the issue first for a variety of reasons including lack of time, lack of knowledge about sexual health, worry about causing offense, and personal discomfort [48-51]. Yet patients largely report that they would not be offended by sexual health questions and would like their providers to ask [52] or are okay with providers initiating sexual health conversations, even when they have a preference for starting the discussion themselves [53]. In a recent qualitative study, a general practitioner in a French outpatient clinic asked 93 patients about their sexual health, tying the question to a presenting concern or asking at the end of the appointment [54]. A majority (92%) of patients expressed positive (31%) or neutral (61%) feelings about the sexual health question when the provider followed up with a question about the patient’s reaction to the question.

Studies continue to find that patients have unvoiced sexual health concerns that go unassessed [55,56]. A study using nationally representative samples of heterosexual and lesbian, gay, and bisexual Americans underscores that, while collecting SOGI data is vitally important, it is not enough [12]. Across sexual orientation groups, the percentage of respondents who reported talking with their provider about sexual health over the past year was 8% to 15% (no significant between-group differences). This contrasts with the 22% to 42% of respondents who reported experiencing a persistent sexual health concern in the past year. This demonstrates a gap between the prevalence of sexual health concerns and the extent to which they are addressed.

### Technological Barriers

A major barrier for VHA in assessing SOGI and sexual health has been the lack of structured data fields for this information within the current electronic health record (EHR) [57]. The VHA’s current EHR has no national sexual health assessment template or note title. This means that, even when providers collect SOGI or sexual health information, these data cannot be readily accessed by other providers. Health care systems’ use of standardized templates or easily identifiable sexual health notes will facilitate providers’ assessment and documentation of patients’ sexual health. When one VHA postdeployment clinic included sexual health in their standard intake, this information was collected and more readily accessible. Researchers examining these intakes found that between 17% to 24% of men assessed in this clinic expressed concerns about their sexual health [58-60].

Whether a barrier to sexual health assessment is poor patient-provider communication or a lack of technological capacity in an EHR system, these barriers will need to be overcome. Fortunately, VHA is moving ahead on several initiatives to reduce these barriers. These initiatives could inform similar implementation efforts in other health care systems.

## Overcoming Barriers to Sexual Health Assessment

### VHA’s Health Record Modernization

Several opportunities exist for VHA to improve the sexual health assessment of veterans. Notably, VHA will soon have SOGI data fields within its current EHR. Plans to permit veterans to enter and edit SOGI data themselves through a web portal prior to an initial visit to establish care are currently underway. VHA has also developed a national sexual health note template for providers to facilitate assessment and tracking. In addition, VHA is replacing its current system with a commercial EHR product (Cerner Millennium, Sweden). Cerner Millennium has SOGI fields and three sexual health screening modules that will improve collecting and tracking this information.

The three standardized sexual health assessment forms in VHA’s Cerner Millennium include a brief history screen designed for intake or hospital admission (eg, sexual concerns to discuss with the provider). The second module is a brief sexual health risk screen that focuses on history of and efforts to prevent STIs. The third module is a more detailed sexual health risk assessment that asks about changes in sexual frequency, desire, satisfaction, and function, and evidence of pain or coercion to engage in unwanted sexual activity. Together, these assessments provide structure to make it easier for VHA providers to use best practices when assessing sexual health and, crucially, give room for patient-centered care that goes beyond a focus on risk and dysfunction [47,61,62]. The national sexual health template for the current VHA EHR combines all three assessments from Cerner Millennium. The template will also pull in basic patient data, including gender identity and sexual orientation when these data are available. If not available, the provider is prompted to ask about these identities.

### Provider Education

Education programs will follow implementation of the new SOGI fields in the current EHR and the expansion of Cerner Millennium to additional facilities. Two trainings on gender identity—one for current EHR users and one for Cerner Millennium users—have already been released. Separate trainings on sexual orientation and on sexual health (for current EHR users and for Cerner Millennium users) are nearing completion. Training will support all members of primary care teams and patients in recognizing the link between sexual health, general health, and well-being [63-65]. These trainings will also help providers learn how to incorporate and document SOGI and sexual health assessment into their workflows. Educational interventions will include resources for primary care teams to help them conduct sexual health assessments.

Table 1 is an example of a brief sexual health assessment that could be adapted to a “pocket card” format. Providers following this guide would first collect SOGI information, including a 2-step sex assigned at birth (confirming information in the record system) and gender identity assessment. They would then conduct a brief sexual health assessment using the “Five ‘P’” model developed by the Centers for Disease Control and Prevention (CDC) with additional questions incorporating a sixth “P” for pleasure [3,61]. The questions in Table 1 were adapted from a CDC guide, the Cerner Millennium modules being implemented in VHA, and other articles discussing sexual health assessment [62]. Table 2 lists all the questions and responses contained in the Cerner Millennium modules being implemented within VHA. While Table 2 is too large for a “pocket card,” the modules could be broken into separate cards and given to primary care team members assigned to complete those modules. Brief role-plays during preclinic stand-ups could help provider teams become comfortable using these tools.

Teams will be encouraged to assign roles and responsibilities for completing the sexual health intakes and following up on identified concerns. While general recommendations focus on increasing the use of nursing staff [13,66], the VHA’s transition to using Patient Aligned Care Teams in primary care makes it well situated to implement sexual health assessments conducted by several team members, each responsible for completing different components. In addition, as the VHA has also committed to increasing access to whole health coaching, whole health coaches could also play an important role in completing aspects of the sexual health assessment.

**Table 1:** Sexual orientation and gender identity, and 6 P’s pocket card.

			Questions
**Sexual orientation and gender identity**			
	Sexual orientation	What is your sexual orientation?	
	Sex at birth	What was your sex assigned at birth?	
	Gender identity	What is your gender identity?	
Global			Do you have any sexual health questions or concerns?
**6 P’s**			
	Partners	Have you been sexually active in the last 12 months? In the past 12 months, how many people have you had sex with? In the past 12 months, how many of your sexual partners have been new partners for you? What genders do your partners identify with?	
	Practices	What parts of your body are involved when you have sex? Have you exchanged sex for your needs (money, housing, drugs, etc)?	
	Protection from STIs^a^	What do you do to prevent STIs? Have you been vaccinated for human papillomavirus, hepatitis A, or hepatitis B?	
	Past history of STIs	Have you been tested for STIs in the past? Would you like to be tested? Have you been diagnosed with an STI in the past? Have any of your partners been diagnosed with an STI?	
	Prevention of pregnancy	How important is it to you to prevent pregnancy? Are you or your partner using contraception or practicing any form of birth control?	
	Pleasure	How satisfied are you with your or your partners’ sexual functioning? Has there been any change in your or your partners’ sexual desire or the frequency of sexual activity? Do you or your partners use any particular devices or substances to enhance your sexual pleasure?	

^a^STI: sexually transmitted infection.

**Table 2:** Veterans Health Administration Cerner pocket cards.

		Response options
**Sexual orientation and gender identity**		
	Confirm sex assigned at birth as listed in the health record. What is your current gender identity (check all that apply)?	Male, female, nonbinary, transgender male, transgender female, does not wish to disclose, other
	Preferred pronouns	He/him/his, she/her/hers, they/them/theirs, ze/zin/zirs, patient name, other
	Do you think of your sexual orientation as...	Straight or heterosexual; lesbian, gay, or homosexual; bisexual; something else, please specify (select other); do not know; choose not to disclose; other
**Sexual health history screen**		
	Do you have any sexual health “questions or concerns” that you would like to discuss with your provider?	Yes, no
	Have you been sexually active in the last 12 months?	Yes, no
	If no, have you ever been sexually active?	Yes, no
	If yes, in the past 12 months, how many people have you had sex with?	[Numeric]
	In the past 12 months, how many of your sexual partners have been new partners for you?	[Numeric]
	In the last 12 months, have your sexual partners been...	Male, female, both
**Sexual health risk screening**		
	Have any of your partners in the past 12 months ever been diagnosed with a sexually transmitted infection?	Yes, no
	Have any of your sexual partners in the last 12 months had HIV?	Yes, no
	Have any of your sexual partners in the last 12 months injected drugs?	Yes, no
	Have you exchanged sex for money, drugs, or other nonmonetary items in the last 12 months (transactional sex)?	Yes, no
	What are you doing to prevent sexually transmitted infections?	Abstinent (choosing not to have sex), reduce number of sexual partners, maintains monogamous relationship (only one partner), uses condoms or other barrier methods, other
	Do you have any sexual health questions or concerns that you would like to discuss?	[Open response]
**Detailed sexual health history assessment**		
	How satisfied are you with you (or your partner’s) sexual functioning?	[Open response]
	Has there been any change in your (or your partner’s) sexual desire or the frequency of sexual activity?	[Open response]
	Do you (or your partners) use any particular devices or substances to enhance your sexual pleasure?	Yes, no
	What kinds of devices or substances?	Other
	Do you ever have pain with intercourse?	Yes, no
	Do you have any difficulty with lubrication?	Yes, no
	Do you have any difficulty achieving orgasm?	Yes, no
	Do you have any difficulty obtaining and maintaining an erection?	Yes, no
	Do you have difficulty with ejaculation?	Yes, no
	Is there anything about your (or your partner’s) sexual activity (as individuals or together) that you would like to change?	Yes, no
	What other concerns or questions regarding your sexual health or practices would you like to discuss (eg, pain, low/high sex drive, or safe practices)?	[Open response]

### Patient Education

In the VHA, a veteran education campaign with brief public service announcements and on-site information will also be needed to inform patients that they will be asked about their SOGI and sexual health and why. When self-report mechanisms are available, veterans will need to be informed about how to use them and how their information will be used and protected. Fact sheets for veterans on why gender identity is asked are already available. Fact sheets for veterans on why sexual orientation and sexual health are assessed are nearing completion. These fact sheets will be available at VHA facilities and on VHA websites. In addition, VHA could leverage its expertise in developing phone apps to create a sexual health education tool targeted toward adult patients. Currently, there is a dearth of apps providing comprehensive information about sexual health [67]; a well-designed app would likely benefit veterans both inside and outside the VHA.

The evidence we reviewed points to the gap in patient-provider communication about sexual health largely due to provider perception. This appears to be driven by providers’ beliefs that patients are not willing to discuss their sexual health when, in fact, they are. While veteran educational interventions are important, provider education is critical for promoting sexual health assessment.

## Conclusion

While sexual health is an important part of overall health, health care providers do not routinely assess patients’ sexual health. The primary barriers seem to be providers’ beliefs that patients will be offended if asked about sexual health and logistical barriers to assessing sexual health. We believe that adding sexual health assessments directly to EHRs and pairing these changes with provider education about using these tools will help. Focusing provider education on addressing the belief gap between providers and patients (ie, sharing evidence that patients are largely comfortable with sexual health assessment) and interventions to increase providers’ comfort with discussing sexual health will likely increase the impact of logistical changes. In addition, focusing on patients’ priorities and sexual well-being could potentially increase patient engagement in care and enhance the whole health of our patients.

Finally, most research on sexual health has focused on risk, dysfunction, and treatment. Research is needed to identify commonly held sexual health goals and promote healthy sexual functioning. Implementing routine sexual health assessment and providing structural support for providers to do so and to ensure the information is accessible to the entire care team would make such research more feasible. This approach could move health care from reacting to problems to preventing them and to promoting healthy sexual functioning for optimal health.

## Abbreviations

CDC: Centers for Disease Control and Prevention
EHR: electronic health record
LGBTQ+: lesbian, gay, bisexual, transgender, queer, and similar minority
SOGI: sexual orientation and gender identity
STI: sexually transmitted infection
VHA: Veterans Health Administration
